# Immune Checkpoint Inhibitors-Associated Thrombosis: Incidence, Risk Factors and Management

**DOI:** 10.3390/curroncol30030230

**Published:** 2023-03-04

**Authors:** Tzu-Fei Wang, Marc Carrier

**Affiliations:** Department of Medicine, University of Ottawa at The Ottawa Hospital and Ottawa Hospital Research Institute, Ottawa, ON K1H 8L6, Canada

**Keywords:** immune checkpoint inhibitors, venous thromboembolism, cancer-associated thrombosis, malignancy, anticoagulation, thrombosis

## Abstract

Immune checkpoint inhibitors (ICIs) target programmed cell death (PD) 1 receptor and its ligand PD-L1, and have become an integral part of treatment regimens in many cancers including lung cancer, renal cell carcinoma, melanoma, and more. Cancer is associated with a significantly increased risk of venous thromboembolism compared to non-cancer patients, and the risks increase further with anticancer therapies including ICIs. Cancer-associated thrombosis can lead to hospitalizations, delayed cancer treatment, and mortality. While thrombosis was not reported as a major complication in initial clinical trials leading to the approval of ICIs, emerging evidence from post-marketing studies revealed concerning risks of thrombosis in patients receiving ICIs. However, results remained heterogenous given differences in study designs and populations. Recent studies also showed that C-reactive protein dynamics might be an easily accessible biomarker for thrombosis and disease response in this population. In addition, early findings indicated that a commonly used anticoagulant for cancer-associated thrombosis, factor Xa inhibitors, might have potential synergistic antitumor effects when combined with ICIs. Herein we will review the current literature on the incidence, risk factors, and management of thrombosis in patients with cancer receiving ICIs. We aim to provide valuable information for clinicians in managing these patients.

## 1. Introduction

Tumor cells evade immune destruction by activating immune checkpoint receptor proteins including cytotoxic T-lymphocyte-associated protein 4 (CTLA-4) and programmed cell death protein 1 (PD-1) found on T cells, and programmed death ligand 1 (PD-L1) found on tumor cells. A type of novel anticancer therapy termed “immune checkpoint inhibitors (ICIs)” involves monoclonal antibodies that specifically target these proteins and prevent immune escape from tumor cells. Since the approval of the first ICI (ipilimumab) in 2011, seven ICIs have been approved in Canada and the United States in approximately 20 different malignancies (Table 1), including ipilimumab (anti-CTLA-4), nivolumab, pembrolizumab, cemiplimab (anti-PD-1), and atezolizumab, durvalumab, avelumab (anti-PD-L1). As such, patients eligible for ICI treatment significantly increased from 1.5% in 2011 to 43.6% in 2018 [1]. Sustained responses and significant improvement in survival have been seen with these novel therapies, and they have become the mainstay of therapies in many cancers such as melanoma, lung cancer, renal cell carcinoma (RCC), and more [2]. 

Although thrombosis was not raised as a major concern in initial randomized controlled trials (RCTs) leading to the approval of ICIs, reports of thromboembolic disease had emerged from post-marketing wide use of these agents. Cancer-associated thrombosis is a known phenomenon, and it is estimated that 20% of cancer patients will develop thrombosis during their cancer journey [3]. It can result in hospitalizations, delayed cancer treatment, significant morbidity, and mortality [4,5]. Patients often experience psychosocial and financial distresses from the diagnosis of thrombosis as well [6,7]. Therefore, the optimal prevention and treatment strategies for cancer-associated thrombosis are crucial in the care of cancer patients. Our previous review demonstrated that the reported rate of venous thromboembolism (VTE) was 5-8% in 6 months and over 10% in 12 months in patients receiving ICIs [8]. More studies have been published since then, providing new insights in the incidence, biomarkers, and predictors of thrombosis in this population. Herein we aim to provide an updated narrative review on thrombotic complications in patients with cancer receiving ICIs. For this review, we researched the MEDLINE database from inception to January 31, 2023 by using terms including (“thrombosis” OR “thromboembolism”) AND (“cancer “OR “malignancy”) AND (“immune checkpoint inhibitors” OR “immunotherapy”) and reviewed all relevant prospective or retrospective cohort studies, systematic reviews and meta-analyses, review articles, as well as conference proceedings. We then summarized key information from the literature herein. 

## 2. Mechanism of Thrombosis and Immunotherapy

**Figure 1:**
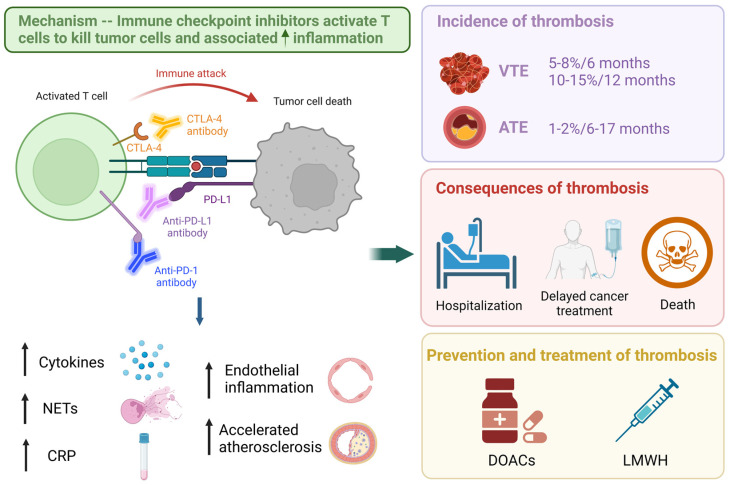
Summary of mechanism, incidence, consequences, prevention, and treatment of immune checkpoint inhibitor-associated thrombosis. Figure was created with BioRender.com and adapted from “Immune Checkpoint Inhibitor Against Tumor Cell”, by BioRender.com (2023). Retrieved from https://app.biorender.com/biorender-templates (accessed on 2 February 2023).

While the exact mechanisms of ICI-associated thrombosis remain unclear, studies have made strides to understand them. Programmed cell death protein 1 is crucial in downregulating pro-atherogenic T cell responses, so PD-1 antibodies can exacerbate atherosclerotic inflammatory vascular lesions [9] (Figure 1). Studies have shown that ICIs are found to be associated with increased T cell activation and endothelial inflammation, leading to thrombosis formation and accelerated atherosclerosis [10,11]. Similar to other immune-related adverse events associated with ICIs such as colitis and pneumonitis, ICIs release the break on immunoregulatory pathways, lead to an increase in inflammation and related cytokines, activate blood and endothelial cells, as well as release of neutrophil extracellular traps (NETs), and eventually result in thrombosis [12].

## 3. Incidence of Thrombosis in Patients Receiving Immune Checkpoint Inhibitors

Initial RCTs investigating the efficacy of ICIs as anticancer treatment did not report thrombosis as a major adverse event of concern. An initial meta-analysis of these RCTs showed a modest rate of VTE at 2.7% (95% confidence interval [CI] 1.8–4.0) and an arterial thrombosis (ATE) rate of 1.1% (95% CI 0.5–2.1) [13]. Another meta-analysis of RCTs and prospective studies of ICI use in patients with melanoma and non-small cell lung cancer (NSCLC) also reported similar rates (VTE rates: 1.5% in melanoma and 1.9% in NSCLC) [14]. Most recently, an updated meta-analysis showed no significantly increased risk of VTE (odds ratio [OR] 0.99, 95% CI 0.82–1.19) in patients treated with ICIs compared to non-ICI regimens [15]. However, all of these meta-analyses suffered from the possibility of underreporting of thrombosis events in RCTs in the first place. Solinas et al. excluded 40% of the eligible RCTs from analysis as they did not specifically report rates of thrombosis [13]. In addition, in these studies, thrombosis events were reported as adverse events based on the Common Terminology Criteria for Adverse Events (CTCAE) criteria instead of planned outcomes, and adverse events were often only reported if they were over certain percentages. Significant underreporting of thrombosis events had been shown in previous oncology RCTs with focus on efficacy of cancer therapies compared to thromboprophylaxis trials, which requires close attention for data interpretation [16]. 

Since the approval and wide clinical use of various ICIs, occurrences of venous and/or arterial thromboses are increasingly reported. Table 2 summarized the results of 27 cohort studies reported to date, with the majority being retrospective cohorts from single centers. Most (80–100%) patients had metastatic cancer, with the most common cancers including NSCLC, melanoma, and RCC. All studies included a variety of different ICIs available in clinical care. The incidence of thrombosis differed widely among studies, due to diverse types of underlying malignancies, concurrent cancer therapies (such as chemotherapy), and variable follow-up durations. In general, the incidence of VTE was 5–8% at 6 months and 10–15% at 12 months (Table 2). Overall, the rates reported in these retrospective cohort studies were higher than those reported from RCTs (1–2% in meta-analyses) [13,14], but not considerably higher when considering the 6-month risks of VTE of 9–10% in ambulatory cancer patients with Khorana score of ≥ 2 receiving chemotherapy [17]. However, given the efficacy of ICIs, the exposure of ICIs can be prolonged, and the risks of thrombosis can continue to accumulate. Sheng et al. showed that the thrombosis rate did not plateau until approximately 30 months in patients with metastatic RCC and 36 months in those with metastatic urothelial cancer [18,19]. Therefore, as patients continue ICIs, the risks of thrombosis can continue to increase. This is worth noting as compared to chemotherapy, for which the highest risk of thrombosis is typically seen within the first 6 months. 

In addition to retrospective cohort studies, three Danish population cohort studies reported the incidence of thrombosis in patients on ICIs (Table 3) [43,44,45]. It is interesting to note that the rates of thrombosis appear to be numerically lower in the population studies compared to the above-mentioned retrospective studies, with 6-month VTE rate of 2–4% and 4–7% at 12 months. In these population studies, outcomes were identified by ICD10 codes +/− imaging codes (as compared to individual chart review in retrospective studies), which could account for the difference in event rates. In addition, all three studies were from a single country (Danish registry), as compared to various countries from cohort studies (Table 2).

Whether ICI combinations or combined ICI and chemotherapy would be associated with an increased risk of VTE (compared to single ICI or chemotherapy alone) is also an area of debate. Sussman et al. showed that ICI combinations were associated with a higher risk of VTE compared to single agent ICI [46]. A recent meta-analysis also showed that compared to mono-ICI, combined ICIs were associated with an increased risk of VTE and myocardial infarction in those with NSCLC [14]. However, another study showed that combined ICIs were not associated with higher risks [47]. Similarly, some studies showed an increased risk of ICI–chemotherapy combination compared to chemotherapy alone [31,42], while others showed similar rates of thrombosis in patients receiving ICI alone, chemotherapy alone, or combination [22]. Most recently, in the analysis of an oncology database of 2299 patients with stage IV NSCLC, first-line ICI-based regimens were associated with a 26% reduction in the risk of VTE compared to chemotherapy-based regimens, while the risks were comparable between ICI/chemo versus chemotherapy alone [40]. It is worth noting that a fair comparison of the risks of thrombosis associated with combination of chemotherapy and ICIs to ICI alone or chemotherapy alone could be challenging, as the baseline characteristics of these patients commonly differ significantly. 

Data on the incidence or arterial thrombosis were even more scant, with rates within 1-2% over a follow-up period of 6 to 17 months (Table 2). A recent meta-analysis showed an increased risk of arterial thrombosis (OR 1.58, 95% CI 1.21–2.06) associated with ICI regimens [15]. In a matched cohort study of 2842 patients, Drobni et al. revealed that there was a three-fold increased risk of cardiovascular events after the start of ICIs compared to other anticancer therapies. The risks were similarly increased when they compared before and after ICI use in the same patients [25]. They also found ICI to be associated with a > three-fold higher rate of progression of total aortic plaque volume, which can be attenuated with concomitant use of corticosteroids or statins [25]. 

## 4. Biomarkers for Thrombosis in Patients on Immune Checkpoint Inhibitors

C-reactive protein (CRP) dynamics was recently identified as a useful biomarker to predict treatment responses associated with ICIs. Significant improvement of objective response, progression-free survival (PFS), and overall survival (OS) had been shown in patients who were CRP flare-responders (CRP levels more than doubled compared to baseline within one month of ICI initiation and subsequently dropped to lower than baseline within 3 months) [48,49]. Therefore, the use of early CRP dynamics as a biomarker in predicting ICI-associated VTE was explored [50]. In a retrospective study of 405 patients receiving ICIs, CRP was measured at baseline within 4 weeks prior to ICI initiation and longitudinally (every 4 weeks) within the first 3 months after ICI initiation. CRP flare was defined as an increase of CRP by a factor of 2.5, while CRP response was defined as a 50% reduction of CRP. In the multivariable analysis accounting death as a competing risk, early CRP flare was associated with a hazard ratio (HR) of VTE of 3.58 (95% CI 1.07–11.94) compared with no CRP flare. Patients with CRP response with no prior flare had the lowest risks of VTE. VTE was independently associated with mortality in those with CRP flare. This study indicated the role of inflammation in the pathophysiology of ICI-associated VTE and revealed a readily available biomarker that has a good potential to predict VTE in this population. Pending further validation, CRP can become a useful tool in this setting. 

In another study, blood samples from 25 patients at the time of starting ICIs (15 subsequently developed VTE and 10 did not) were analyzed and showed that patients who subsequently developed VTE had a significant elevation in the numbers of total myeloid-derived suppressor cells (MDSCs) and levels of inflammatory cytokines including ginterleukin-8 (IL-8), IL-1 receptor antagonist, soluble vascular cell adhesion molecule 1 (sVACM-1), and granulocyte–macrophage colony stimulating factor (GM-CSF) [47]. These markers shed light on the mechanism of thrombus formation in patients treated with ICIs and again indicated the importance of immune-mediated inflammation in the process. 

As for ICI-associated arterial thrombosis, a small study of 30 patients on ICIs showed that high sensitivity (hs)-troponin T (TnT) ≥ 14 ng/L was associated with a higher risk of cardiovascular outcomes (including cardiovascular death, stroke, transient ischemic attacks, pulmonary embolism, and/or new onset heart failure) [51]. Another retrospective study of 135 patients with metastatic cancer on first-line pembrolizumab revealed that after a mean follow-up of 490 days, hs-troponin I (TnI) > 50 ng/L prior to the first and the second dose of pembrolizumab was an independent predictor of major adverse cardiac events (MACE) (including myocarditis, acute coronary syndrome, heart failure, VTE, cardiovascular hospitalization, and/or mortality) (HR 8.1, 95% CI 1.67–37.4) [52]. hs-TnI > 50 nl/L prior to first dose of pembrolizumab was also associated with increased all-cause mortality. Given these findings, the 2022 European Society of Cardiology (ESC) guidelines on cardio-oncology recommended to monitor cardiac troponin prior to each cycle of ICIs [53].

## 5. Risk Factors of Thrombosis in Patients on Immune Checkpoint Inhibitors

Understanding risk factors for thrombosis in cancer patients can assist identification of high-risk patients and tailor thromboprophylaxis accordingly. Many risk prediction models had been proposed, among which Khorana score is the most extensively validated. Khorana score was initially derived and validated in 4066 patients enrolled in the Awareness of Neutropenia in Chemotherapy (ANC) Study Group Registry in the United States and had been subsequently validated in many external cohorts including over 30,000 ambulatory cancer patients [54,55]. It includes several easily identified variables in cancer patients undergoing chemotherapy, including the type of malignancy, body mass index ≥ 35 kg/m^2^, pre-chemotherapy leukocyte count > 11 × 10^9^/L, hemoglobin < 10 g/dL, and platelet count ≥ 350 × 10^9^/L [54]. Recent RCTs confirmed the utility of Khorana score to stratify risks of cancer-associated thrombosis and target thromboprophylaxis in ambulatory cancer patients with Khorana score ≥ 2 (intermediate-high risk) [56,57]. Therefore, although the score has some limitations, it is endorsed by major guidelines to use for risk stratification in ambulatory cancer patients [58,59]. As the development of the Khorana score predated ICIs, whether it can accurately predict thrombosis in patients receiving ICIs remains controversial as studies have shown mixed results. Most studies did not find the Khorana score to carry sufficient predictivity in this population [18,23,29,31,32,33,35,36,37], while a few studies did [27,45,46]. 

In addition to the Khorana score, many studies attempted to identify additional risk factors for thrombosis in this population. However, risk factors found varied significantly among studies, as most studies were single-center with small sample sizes (Table 4). Potential risk factors identified included (1) patient-related factors, such as female gender [20,28], history of thromboembolism [22,24,27,35,37,38,39,46], younger age [26,27,47], smoking [23,26], and poorer ECOG status [29,39]; (2) cancer-related factors, such as lung cancer [22,25], and metastasis [36,37,47]; and (3) treatment-related factors, such as combined ICI use [46], combined chemotherapy and ICI use, and more. A specific risk prediction model in this population to help identify patients at high risk who could benefit from primary thromboprophylaxis will be of interest. 

As for arterial thrombosis, evidence is scant, but few available studies identified risk factors not different from traditional risk factors for arthrosclerosis, such as age, diabetes, hypertension, smoking, history of cardiovascular disease (Table 4). 

## 6. Consequences of Thrombosis in Patients on Immune Checkpoint Inhibitors

Thrombosis can lead to hospitalizations, delay in cancer treatment, and other morbidity and mortality. In a study of 351 patients with metastatic RCC receiving ICIs, 12% (n = 43) developed thrombotic events over a median follow-up of 12.8 months, and 72% of these events resulted in hospitalizations, with 21% having a median delay of 14 days in subsequent ICI doses [18]. In another study of 279 patients with metastatic urothelial cancer, thrombotic events resulted in 83% hospitalization, and 7% delayed ICI treatment with one discontinuation of ICI due to thrombosis [19]. 

In addition, worsening survival has been shown in patients with thrombosis (either VTE or combined venous and arterial thrombotic events) in this population in many studies [18,19,35,36,37,38,46], while some other (but fewer) studies did not show such an association [26,28,31]. The discrepancy could be due to factors such as patient population, type, and status of cancer. 

## 7. Prevention and Treatment of Thrombosis in Patients on Immune Checkpoint Inhibitors

Recent RCTs including AVERT and CASSINI trials demonstrated the efficacy and safety of prophylactic doses of apixaban and rivaroxaban, respectively, in the prevention of cancer-associated thrombosis in ambulatory cancer patients with intermediate-high risk patients (Khorana score ≥ 2) [56,57]. Major international guidelines have since suggested consideration of primary VTE prophylaxis in this population [58,59]. However, whether this practice can also apply to patients receiving ICIs remains unclear, as Khorana score, derived from a chemotherapy-treated population, had been shown to be suboptimal in risk stratification for patients on ICIs [18,23,29,31,32,33,35,36,37]. Patients with Khorana score of ≥ 2 on chemotherapy are associated with a 6-month risk of VTE of 9–10% [17], and whether the ICI-treated population with a 6-month VTE risk of 5–8% can derive sufficient benefit to warrant primary thromboprophylaxis in all patients receiving ICIs remains investigational. Further identification of high-risk patient population may be needed. 

Treatment for cancer-associated VTE in patients receiving ICIs is usually not different from all other cancer-associated thrombosis. Anticoagulation is the mainstay of treatment, with direct oral anticoagulants and low-molecular-weight heparin (LMWH) as the most commonly used anticoagulants in the cancer population; the choice of anticoagulants should take into account patient characteristics, tumor characteristics, risk of bleeding, patient preference, and affordability [59,60].

## 8. Anticoagulation and Cancer Survival 

Preclinical studies showed that coagulation factors such as factor X could assist tumors to escape the immune system, thus contributing to resistance of ICIs. In mice models, factor Xa inhibitors such as rivaroxaban could augment the effects of ICIs against tumor cells and inhibit tumor growth [61,62]. Therefore, whether this can translate into clinical practice—to use factor Xa inhibitors to enhance the antitumor effects of ICIs—is of particular interest. A retrospective study disappointingly showed no difference in response rates, PFS, or OS in patients receiving ICIs and therapeutic anticoagulation compared to those without anticoagulation [63]. However, the anticoagulants used in the study were not limited to factor Xa inhibitors and there was imbalance in baseline characteristics in those who were treated on anticoagulation [63]. In a more recent German study of 280 patients with stage IV melanoma treated with ICIs, again there was no significant difference in the overall response rate, disease control rate, PFS, or OS comparing patients on anticoagulation to those who were not (after adjusting for baseline characteristics differences) [30]. However, when stratified by the type of anticoagulant, the 27 patients who received factor Xa inhibitors had a significant improvement in PFS compared to those on other anticoagulants (median PFS 12 vs. 2 months; HR 0.36, 95% CI 0.2–0.64) and those not on anticoagulants (median PFS 4 months). The OS, as well as overall response rate and disease control rates, were all significantly superior with concomitant factor Xa inhibitors. The improvements in outcomes were not explained by the differences in baseline characteristics. Reassuringly, there were no differences in bleeding events (major and clinically relevant-non major bleeding) among patients receiving factor Xa inhibitors compared to those not on anticoagulation or on different types of anticoagulants during follow-up. The proposed mechanism is that factor Xa inhibitors can penetrate the tumor microenvironment to inhibit signaling function of macrophage-derived factor Xa that promote immune evasion, while LMWH depends on antithrombin for function and is restricted to intravascular space, and thus could not provide synergistic effects to ICIs as factor Xa inhibitors. This is the first study to provide clinical evidence that there could be a synergy between factor Xa inhibitors and ICIs and requires further validation. If confirmed in larger studies, factor Xa inhibitors can play a potential role in optimizing ICI treatment. 

## 9. Conclusions 

Immune checkpoint inhibitors are increasingly used and with prolonged duration. Recent studies have shown that rates of VTE in patients receiving ICIs appeared higher than initially reported in RCTs but might be similar or not as high as those receiving chemotherapy. However, the risk can continue to increase with continued ICI use. Arterial thrombosis should also be considered in this population. It is crucial to understand thrombosis as an important adverse event for the optimal management of these patients. The potential synergistic antitumor effects of factor Xa inhibitors and ICI is exciting, but further investigation is needed. 

## Figures and Tables

**Table 1:** Summary of approved immune checkpoint inhibitors (and their indications).

Immune Checkpoint Inhibitors	Target	Approved Indication
Ipilimumab	CTLA-4	Melanoma NSCLC RCC Colorectal cancer Malignant pleural mesothelioma
Pembrolizumab	PD-1	Melanoma NSCLC Urothelial carcinoma RCC Bladder cancer Esophageal/esophagogastric junction cancer Colorectal cancer Endometrial cancer Cervical cancer Breast cancer Head and neck squamous cell carcinoma Hodgkin lymphoma Primary mediastinal B cell lymphoma
Nivolumab	PD-1	Melanoma NSCLC RCC Head and neck squamous cell carcinoma Classical Hodgkin lymphoma Hepatocellular carcinoma
Cemiplimab	PD-1	NSCLC Cutaneous squamous cell carcinoma Cutaneous basal cell carcinoma Cervical cancer
Atezolizumab	PD-L1	NSCLC Small cell lung cancer Urothelial carcinoma
Avelumab	PD-L1	Urothelial carcinoma Merkel cell carcinoma
Durvalumab	PD-L1	NSCLC Urothelial carcinoma

Mostly approved for unresected or metastatic cancers, refer to individual monographs for detailed approval indications for each cancer. Abbreviations: NSCLC: non-small cell lung cancer; RCC: renal cell carcinoma.

**Table 2:** Summary of incidence rates of venous and arterial thrombosis from retrospective studies of cancer patients receiving immune checkpoint inhibitors (studies missing follow-up durations were not included).

Study	Country	N	Type of Cancer	Stage IV	Follow-up [Median (IQR)]	VTE Incidence % (95% CI)	ATE Incidence % (95% CI)
Hegde et al. 2017 [20] Abstract	USA	76	Lung	N/A	10.8 mo	18.4	2.6
Ibrahimi et al. 2017 [21] Abstract	USA	154	Lung 20.8% Melanoma 20.1% Ovarian 12.3%	92%	7 mo (198 days)	10.4	0
Bar et al. 2019 [22]	Israel	1215	All cancers Melanoma 40.5% Lung 28.7%	N/A	12 mo	AVE (MI, stroke, PE, DVT) 6 mo: 4.9 12 mo: 5.8	
Nichetti et al. 2019 [23]	Italy	217	NSCLC	95.4%	37.8 mo	7.4	6.5
Ando et al. 2020 [24]	Japan	122	Lung, kidney, stomach, urothelial, melanoma	N/A	N/A Time to thrombosis 90 days (range 6–178)	4.1	4.9
Drobni et al. 2020 [25]	USA	2842	All cancers NSCLC 28.8% Melanoma 27.9%	N/A	2 years	N/A	Composite: 5.35/100 person-yrs MI: 2.49 Stroke: 2.08
Deschênes-Simard et al. 2021 [26]	Canada	593	NSCLC	87.2%	12.7 (4.9–22.7) mo	9.9 (7.5–12.3) 76.5 (59.9–97.8) per 1000 person-years	1.3
Gong et al. 2021 [27]	USA	2854	All cancers NSCLC 28.4% Melanoma 28.2%	N/A	194 days (IQR 65–412)	6 mo: 7.4 12 mo: 13.8	N/A
Gutierrez-Sainz et al. 2021 [28]	Spain	229	Lung 48% Melanoma 23.6% RCC 11.8%	96.5%	9.8 mo	7 (4–10)	N/A
Guven et al. 2021 [29]	Turkey	133	RCC 26.3% Melanoma 24.1% NSCLC 18.8%	100%	10.1 (5.8–18.5) mo	11.3	N/A
Haist et al. 2021 [30]	Germany	280	Melanoma	100%	28 mo (95% CI 23.4–32.6)	12.5	4.3
Hill et al. 2021 [31]	USA	435 (a) ICI: 171 (b) ICI+chemo: 157 (c) chemo then durvalumab: 107	NSCLC	47%	N/A	6 mo: (a) 7.6 (4.3–12.2) (b) 9.9 (5.8–15.3) (c) 9.4 (4.8–15.8) 12 mo: (a) 9.0 (5.3–14.0) (b) 12.8 (7.8–19.0) (c) 12.2 (6.8–19.2)	N/A
Icht et al. 2021 [32]	Israel	176	NSCLC	85.8%	6 mo (187 days)	4.5 (2.1–8.3)	N/A
Kewan et al. 2021 [33]	USA	552	All cancers NSCLC 47.3%	100%	12.1 mo	12.1	1.3
Madison et al. 2021 [34] ^	USA	6127	Lung	N/A	6 mo	6.3	2.6
Moik et al. 2021 [35]	Austria	672	Melanoma 30.4% NSCLC 24.1% RCC 11%	85.8%	8.5 mo	6 mo: 5.0 (3.4–6.9) 12mo: 7.0 (5.1–9.3) Overall: 12.9 (8.2–18.5)	6 mo: 1.0 (0.4–2.0) 12 mo: 1.8 (0.7–3.6) Overall 1.8 (0.7–3.6)
Roopkumar et al. 2021	USA	1686	Lung 49.6% Melanoma 13.2%	90.3%	438 days (range 7–1971)	6 mo: 7.1 12 mo: 10.9 Overall: 24	N/A
Sheng et al. 2021 [18]	USA	351	RCC	100%	12.8 mo	11	2
						Total thromboembolism: 6 mo: 4.4 (2.6–6.9) 12 mo: 9.8 (6.8–13.4)	
Sussman et al. 2021	USA	228	Melanoma	81.1%	27.3 mo	6 mo: 8.0 (4.9–12.0) 12 mo: 12.9 (8.9–17.7)	6 mo: 2.2 (0.8–4.8) 12 mo: 4.5 (2.3–7.8)
Alma et al. 2022 [36]	France	481	Lung	86%	9.8 mo	9.8	N/A
Bjornhart et al. 2022 [37]	Denmark	146 prospective (A) *	NSCLC	87%	16.5 mo	6 mo: 13.0 12 mo: 14.4 Overall: 14	N/A
		426 retrospective (B)				6 mo: 4.9 12 mo: 5.6 Overall: 6	
Canovas et al. 2022 [38]	Spain	665	Lung	91.2%	14 mo	6.9	1.5
						All thrombosis: 8.4 (6.23–10.6)	
		291	Melanoma	82.5%	17 mo	4.8	1.0
						All thrombosis: 5.8 (3.34–9.18)	
Endo et al. 2022 [39]	Japan	120	Lung	62.5%	within 6 mo	2.5	4.2
Khorana et al. 2023 [40] ^	USA	(a) ICI: 605 (b) ICI+chemo: 602	NSCLC	100%	9.1 mo	6 mo: (a) 8.1 (b) 12.8 12 mo: (a) 13.5 (10.6–16.5) (b) 22.4 (20.2–24.5)	N/A
May et al. 2022 abstract [41] ^	USA	1823	All cancers	N/A	6 mo	7.3	N/A
Sanfilippo et al. 2022 abstract [42]^	USA	1754	All cancers	77.9%	6 mo	4.1	N/A
Sheng et al. 2022 [19]	USA	279	Urothelial	100%	5.6 mo	13	2
						Total thromboembolism: 6 mo: 9.1 (6.0–13.0) 12 mo: 13.6 (9.6–18.4)	

* A prospective cohort employed screening-detected VTE by computed tomography venography and pulmonary angiography. ^ Outcomes identified by ICD codes. Gong: VTE after ICI compared to pre, HR 4.98 (3.65–8.59). Abbreviations: ATE—arterial thrombosis; AVE—arterial event; CI—confidence interval; DVT—deep vein thrombosis; ICI—immune checkpoint inhibitor; MI—myocardial infarction; mo—months; N/A—not available; NSCLC—non-small cell lung cancer; PE—pulmonary embolism; RCC—renal cell carcinoma; USA—United States of America; VTE—venous thromboembolism.

**Table 3:** Summary of incidence of VTE and ATE from population cohort studies (all from Danish registries).

Study	N	Type of cancer	Follow-up (mo)	VTE (%), (95% CI)	ATE (%), (95% CI)
Mulder et al. 2021 [44]	370	All cancers	6	4.1 (2.3–6.7)	N/A
			12	7.1 (4.2–11.1)	
Moik et al. 2021 [44] Abstract	3259	All cancers	6	3.9 (3.3–4.7)	1.3 (0.9–1.8)
			12	5.7 (4.9–6.6)	2.2 (1.7–2.8)
			24	7.3 (6.2–8.4)	3.1 (2.4–3.8)
Overvad et al. 2022 [45]	3946	All cancers	6	2.6	1.3
			12	3.8	1.9

Abbreviations: ATE—arterial thrombosis; CI—confidence interval; mo—months; VTE—venous thromboembolism.

**Table 4:** Risk factors for thrombosis and mortality identified in patient cohorts receiving immune checkpoint inhibitors.

Study	Risk factors for Thrombosis (Multivariable)	Risk Factors for Mortality
Hegde et al. 2017 [20] (Abstract)	Female	VTE before ICI
Bar et al. 2019 [22]	NSCLC H/o AVE Hypertension Dyslipidemia	AVE
Nichetti et al. 2019 [23]	Current smoker PD-L1 > 50%	TE
Ando et al. 2020 [24]	h/o thromboembolism	N/A
Drobni et al. 2020 [25]	Overall study: ICIs, age, h/o stroke, diabetes, hypertension, NSCLC, male, h/o radiation	N/A
Deschênes-Simard et al. 2021 [26]	Age < 65 Higher PD-L1 level Smoking <12 mo from diagnosis to ICIs	VTE is not correlated with survival
Gong et al. 2021 [27]	Age ≤ 65 Khorana score ≥ 2 h/o hypertension Strong trend: h/o VTE (HR 1.42, 95% CI 0.99–2.06) (melanoma is associated with decreased risks)	N/A
Gutierrez-Sainz et al. 2021 [28]	Female Melanoma	VTE is not an independent risk factor
Guven et al. 2021 [29]	ECOG ≥ 1	VTE (trend, not significant)
Hill et al. 2021 [31]	Cancer treatment types (ICI-chemotherapy, targeted therapies) Smoking	VTE was not associated with significantly worsened survival
Icht et al. 2021 [32]	N/A	VTE
Kewan et al. 2021 [33]	Anticoagulation at the time of ICI (univariate)	Khorana score
Moik et al. 2021 [35]	h/o VTE	VTE after ICI
Roopkumar et al. 2021 [47]	Younger age Metastasis Biomarkers	VTE
Sheng et al. 2021 [18]	None	Thromboembolism, IMDC score, and/or Khorana score
Sussman et al. 2021 [46]	Combination ICI Khorana score ≥ 1 h/o CAD Anticoagulation at treatment start	VTE
Alma et al. 2022 [36]	Metastasis BMI	VTE
Bjornhart et al. 2022 [37]	h/o VTE ICI as first-line treatment Other mets (non-brain, liver, bone)	VTE
Canovas et al. 2022 [38] Lung cancer cohort	Hgb < 10.9 g/dL at the start of ICI NLR < 4.55 h/o thrombosis	Thrombosis
Canovas et al. 2022 [38] Melanoma cohort	LDH > 198 U/L NLR > 3.01	Thrombosis
Endo et al. 2022 [39]	ECOG ≥ 2 and history of thromboembolism	N/A
Khorana et al. 2023 [40]	History of radiation Body mass index ≥ 40 kg/m^2^	N/A
Sanfilippo et al. 2022 [42] (Abstract)	ICI-chemotherapy (vs ICI alone) Severe frailty by CA frailty index	N/A
Sheng et al. 2022 [19]	None	Thromboembolism, Bajorin score 1 and 2

Abbreviations: ATE—arterial thrombosis; AVE—acute vascular events; CAD—coronary artery disease; DVT—deep vein thrombosis; ECOG—Eastern Cooperative Oncology Group; Hgb—hemoglobin; h/o—history of; ICIs—immune checkpoint inhibitors; IMDC: International Metastatic Renal Cell Carcinoma Database Consortium; IQR—interquartile range; KS—Khorana score; MI—myocardial infraction; LDH—lactate dehydrogenase; mo—months; N/A—not available; NLR—neutrophil/lymphocyte ratio; NSCLC—non-small cell lung cancer; PE—pulmonary embolism; TE—thromboembolism; VTE—venous thromboembolism.
